# Caregiver Burden of Family Caregivers of Patients With Dementia in a Shanghai Community

**DOI:** 10.1093/geroni/igaa057.2707

**Published:** 2020-12-16

**Authors:** Juan Li, Zhijian Liu, You Yin

**Affiliations:** 1 Naval Medical University, Shanghai, China; 2 Naval medical University, Shanghai, China; 3 Changzheng Hospital, affiliated to Naval Medical University, Shanghai

## Abstract

This study explored the level of and factors related to Chinese dementia family caregiver burden. We recruited 109 dyads of patients with dementia and their family caregivers from communities in Shanghai, China. Caregiver burden was assessed by Caregiver Burden Inventory. We used multivariate linear regression model to investigate factors associated with caregiver burden, including patients’ socio-demographic information, cognitive function, ADL, neuropsychiatric symptoms, caregivers’ depression, coping strategy, social support and community service utilization. The level of caregiver burden was moderate (65.92±16.74). The patients’ cognitive function and social support were negatively associated with caregiver burden (β=﹣0.850, p＜0.01, β=﹣1.787, p=0.044, respectively). Caregivers’ depression and community service utilization were positively associated with caregiver burden (β=2.109, p＜0.01,β=1.215, p＜0.01, respectively). These findings suggest enhancing social support and reducing depression of caregivers may have the potential of decreasing caregiver burden.

